# Decoding the synaptic dysfunction of bioactive human AD brain soluble Aβ to inspire novel therapeutic avenues for Alzheimer’s disease

**DOI:** 10.1186/s40478-018-0626-x

**Published:** 2018-11-08

**Authors:** Shaomin Li, Ming Jin, Lei Liu, Yifan Dang, Beth L. Ostaszewski, Dennis J. Selkoe

**Affiliations:** Ann Romney Center for Neurologic Diseases, Department of Neurology, Brigham and Women’s Hospital and Harvard Medical School, 60 Fenwood Road, Boston, MA 02115 USA

**Keywords:** Alzheimer’s disease, Amyloid-beta protein, Synaptic plasticity, Long-term potentiation, Oligomers

## Abstract

Pathologic, biochemical and genetic evidence indicates that accumulation and aggregation of amyloid β-proteins (Aβ) is a critical factor in the pathogenesis of Alzheimer’s disease (AD). Several therapeutic interventions attempting to lower Aβ have failed to ameliorate cognitive decline in patients with clinical AD significantly, but most such approaches target only one or two facets of Aβ production/clearance/toxicity and do not consider the heterogeneity of human Aβ species. As synaptic dysfunction may be among the earliest deficits in AD, we used hippocampal long-term potentiation (LTP) as a sensitive indicator of the early neurotoxic effects of Aβ species. Here we confirmed prior findings that soluble Aβ oligomers, much more than fibrillar amyloid plaque cores or Aβ monomers, disrupt synaptic function. Interestingly, not all (84%) human AD brain extracts are able to inhibit LTP and the degree of LTP impairment by AD brain extracts does not correlate with Aβ levels detected by standard ELISAs. Bioactive AD brain extracts also induce neurotoxicity in iPSC-derived human neurons. Shorter forms of Aβ (including Aβ_1–37_, Aβ_1–38_, Aβ_1–39_), pre-Aβ APP fragments (− 30 to − 1) and N-terminally extended Aβs (− 30 to + 40) each showed much less synaptotoxicity than longer Aβs (Aβ_1–42_ - Aβ_1–46_). We found that antibodies which target the N-terminus, not the C-terminus, efficiently rescued Aβ oligomer-impaired LTP and oligomer-facilitated LTD. Our data suggest that preventing soluble Aβ oligomer formation and targeting their N-terminal residues with antibodies could be an attractive combined therapeutic approach.

**Electronic supplementary material:**

The online version of this article (10.1186/s40478-018-0626-x) contains supplementary material, which is available to authorized users.

## Introduction

Alzheimer’s disease (AD) is a neurodegenerative disorder characterized by progressive and irreversible cognitive decline. The pathological hallmarks of AD are the aberrant deposition of extracellular senile plaques comprised of amyloid-beta (Aβ) peptides and intracellular neurofibrillary tangles composed of altered forms of the tau protein. A growing body of evidence from genetic, in vivo imaging and biochemical studies has demonstrated that accumulation of oligomeric, diffusible assemblies of Aβ peptides, rather than mature amyloid fibrils, is the earliest pathogenic event in the ontogeny of AD [7, 43, 62, 70, 71].

Aβ peptides are generated throughout life from the amyloid precursor protein (APP) via a sequential, two-step proteolytic cleavage: first by β-site APP-cleaving enzyme 1 (BACE1), also called β-secretase, and then by the presenilin/γ-secretase complex [36, 49, 80]. Transgenic APP mutant mouse models showed that increasing β- or γ-secretase activity promotes the production of pathogenic Aβ and induces AD-like pathology, while decreasing their activities by inhibitors can reduce brain Aβ levels and ameliorate AD neuropathology and resultant behavioral deficits [9, 13]. Experimentally, the deposition of Aβ can be accelerated in the brains of APP-transgenic mice after intracerebral or intraperitoneal injection with Aβ aggregate-containing brain homogenate. This effect can be prevented by immunodepletion of Aβ from the injected materials, thereby supporting the direct role of Aβ as the seeding agent in this process [12, 40].

Symptomatically, the accumulation of Aβ is thought to play a fundamental role in triggering synaptic dysfunction in neurons and leading to their eventual loss. Synaptic failure is an early event in pathogenesis that can be detected in patients with mild cognitive impairment [2, 60]. Experimentally, soluble Aβ oligomers have been found to selectively block hippocampal long-term potentiation (LTP), widely believed to underlie learning and memory [26, 28, 29, 64, 71]. The impairment of synaptic plasticity can be detected before Aβ deposits in plaques in mouse models of AD [15, 31]. Importantly, active and passive Aβ immunotherapy has been shown to protect against the neuropathology and cognitive deficits observed in APP transgenic models of AD [20, 42] and prevent the soluble Aβ oligomers induced LTP impairment [27, 28].

Over the past two decades, several clinical trials that have attempted to reduce Aβ production by decreasing β- or ɣ-secretase activity or directly targeting Aβ by immunotherapy did not meet pre-specified clinical endpoints in mild to moderate AD patients, perhaps due to the presence of well-established amyloid and tau neuropathology and the complexity of accessing the most pathogenic Aβ species. The specific Aβ species that are most directly related to AD-type neurodegeneration in humans and precede dementia of the Alzheimer type have not been clearly identified. In this regard, numerous studies, including ours, have focused on soluble Aβ oligomers made from synthetic Aβ_40/42_ peptides, secreted by FAD-mutant APP-expressing cells, or isolated directly from human (AD) cortex as to their effects on the hippocampal LTP and long-term synaptic depression (LTD), but the results have been variable and difficult to distil into a central conclusion. Here, we systematically compare the different sources, aggregation states of Aβ as well as various APP fragments that contain Aβ sequences and probe the experimental conditions which can affect the response of hippocampus to the complex array of AD-relevant assemblies.

## Materials and methods

### Mice

The Harvard Medical School and Brigham Women’s hospital Standard Committee on Animals approved all experiments involving mice used for electrophysiology and biochemical assays. All mice (male and female) contained a mixed background of C57Bl/6 and 129. Animals were housed in a temperature-controlled room on a 12-h light/12-h dark cycle and had ad libitum access to food and water.

### Hippocampal slice preparation

Mice (C57BL/6 × 129) were euthanized with Isoflurane at 2 to 3 months of age. Brains was quickly removed and submerged in ice-cold oxygenated sucrose-replaced artificial cerebrospinal fluid (ACSF) cutting solution (in mM) (206 sucrose, 2 KCl, 2 MgSO_4_, 1.25 NaH_2_PO_4_, 1 CaCl_2_, 1 MgCl_2_, 26 NaHCO_3_, 10 D-glucose, pH 7.4, 315 mOsm). Transverse slices (350 μm thickness) from the middle portion of each hippocampus were cut with a vibroslicer. After dissection, slices were incubated in ACSF that contained the following (in mM): 124 NaCl, 2 KCl, 2 MgSO_4_, 1.25 NaH_2_PO_4_, 2.5 CaCl_2_, 26 NaHCO_3_, 10 D-glucose, pH 7.4, 310 mOsm, in which they were allowed to recover for at least 90 min before recording. A single slice was then transferred to the recording chamber and submerged beneath continuously perfusing ACSF that had been saturated with 95% O_2_ and 5% CO_2_. Slices were incubated in the recording chamber for 20 min before stimulation under room temperature (~ 26 °C).

### Electrophysiological recordings

We used standard procedures to record field excitatory postsynaptic potentials (fEPSP) in the CA1 region of the hippocampus. A bipolar stimulating electrode (FHC Inc., Bowdoin, ME) was placed in the Schaffer collaterals to deliver test and conditioning stimuli. A borosilicate glass recording electrode filled with ACSF was positioned in stratum radiatum of CA1, 200~ 300 μm from the stimulating electrode. fEPSP in the CA1 region were induced by test stimuli at 0.05 Hz with an intensity that elicited a fEPSP amplitude 40–50% of maximum. Test responses were recorded for 30–60 min prior to beginning the experiment to assure stability of the response. Once a stable test response was attained, experimental treatments (Aβ species and/or antibodies) were added to the 10 mL ACSF perfusate, and a baseline was recorded for an additional 30 min. For the Anti-Aβ antibodies experiments, the antibodies: 3D6 (3 μg/ml), 266 (3 μg/ml), 82E1 (3 μg/ml), 6E10 (2 μg/ml), 4G8 (1 μg/ml), 2G3 (3 μg/ml), 21F12 (3 μg/ml) or R1282 (3 μg/ml) were added to the AD brain extract aliquots incubated with mixing for 30 min, then adding to the mixture to brain slice perfusion buffer. To induce LTP, two consecutive trains (1 s) of stimuli at 100 Hz separated by 20 s were applied to the slices, a protocol that induced LTP lasting approximately 1.5 h in wild-type mice of this genetic background. To induce LTD, 300 pulses were delivered at 1 Hz. The field potentials were amplified 100x using an Axon Instruments 200B amplifier and digitized with Digidata 1322A. Data were sampled at 10 kHz and filtered at 2 kHz. Traces were obtained by pClamp 9.2 and analyzed using the Clampfit 9.2 program. LTP and LTD values reported throughout were measured at 60 min after the conditioning stimulus unless stated otherwise. Two-tailed Student’s t-test and one-way analysis of variance (ANOVA) were used to determine statistical significance.

### Human brain homogenate preparation

Homogenates of human brains were prepared as described elsewhere [64, 81]. Frozen brain tissue collected at Massachusetts Alzheimer’s Disease Research Center (MADRC Neuropathology Core, Harvard, MA, USA) and Brigham and Women’s Hospital under institutional review board-approved protocols. Frozen samples of temporal or frontal cortex (1 g) were allowed to thaw on ice, chopped into small pieces with a razor blade, and then homogenized with 25 strokes of a Dounce homogenizer (Fisher, Ottawa, ON, Canada) in 4 ml ice-cold 20 mM Tris–HCl, pH 7.4, containing 150 mM NaCl (Tris-buffered saline (TBS)) and protease inhibitors. Water-soluble Aβ was separated from membrane-bound and plaque Aβ by centrifugation at 175,000×*g* and 4 °C in a TLA 100 rotor (Beckman Coulter, Fullerton, CA, USA) for 30 min, and the supernatant (referred to as TBS extract) aliquoted and stored at − 80 °C. The ethical body approving this study was the Partners Institutional Review Board of the Partners Human Research Committee.

### Production of induced neurons (iNs) from human induced pluripotent cells (iPSCs)

The YZ1 iPSC line was obtained from UCONN stem cell core [84] and used to prepare neurogenin 2 (Ngn2)-induced human neurons [86]. iPSCs were maintained in media containing DMEM/F12, Knockout Serum Replacement, pencillin/streptomycin/glutamine, MEM-NEAA, and 2-mercaptoethanol (all from Invitrogen, Carlsbad, CA) with addition of 10 μg/mL bFGF (Millipore, Billerica, MA) directly prior to media application. Neuronal differentiation was performed via doxycycline induced Neurogenin 2 system [86]. iPSCs were plated at a density of 95,000 cells/cm^2^ for viral infection. Lentiviruses were obtained from Alstem with “ultrapure titres” and used at the following concentrations: pTet-O-NGN2-puro: 0.1 μl/ 50,000 cells; Tet-O-FUW-eGFP: 0.05 μl/ 50,000 cells; Fudelta GW-rtTA: 0.11 μl/50,000 cells. To induce Neurogenin 2 expression doxycycline is added on “iN day 1” at a concentration of 2 μg/ml. On iN day 2 puromycin is added at 10 mg/ml and is maintained in the media always thereafter. On iN day 4, cells were plated at 50,000 cells/well on matrigel (BD Biosciences, San Jose, CA)-coated Greiner 96 well microclear plates and maintained in media consisting of Neurobasal medium (Gibco), Glutamax, 20% Dextrose, MEM NEAA with B27, with BDNF, CNTF, GDNF (PeprpTech, Rocky Hill, NJ) each at a concentration of 10 ng/ml. At iN day 14 neurite number and expression of neural markers had reached near maximal levels. Thus, for experiments investigating the effects of AD brain extracts on neuronal viability iNs were used at iN day 21, a time point when iNs were fully mature.

### Addition of AD brain extract to induced neurons (iNs) and live-cell imaging

Production and characterization of human brain extracts and induced neurons (iNs) from human induced pluripotent cells (iPSCs) as described previously [21]. Aliquots (two, 0.5 ml) of mock-immunodepleted (AD) or AW7-immunodepleted brain (ID-AD) extracts were thawed on ice for 30–60 min, vortexed, centrifuged at 16,000 g for 2 min, and buffer exchanged into neurobasal medium supplemented with B27/Glutamax using HiTrap 5 ml desalting column (GE Healthcare, Milwaukee, WI). AD and ID-AD extracts (1 ml) were applied to a desalting column using a 1 ml syringe at a flow rate of ~ 1 ml/min and eluted with culture medium using a peristaltic pump. Then, 0.5 ml fractions were collected. Prior experimentation revealed that the bulk of Aβ eluted in fractions 4 and 5. These two fractions were pooled – this pool is referred to as “exchanged extract”. A small portion (50 μl) of the exchanged extract was taken for Aβ analysis and the reminder used in iN experiments.

Approximately 7 h prior to exchanging AD and ID-AD extracts into culture medium, iN day 21 neurons were placed in an IncuCyte Zoom live-cell imaging instrument (Essen Bioscience, Ann Arbor, MI). Four fields per well of a 96 well plate were imaged every 2 h for a total of 6 h. This analysis was used to define neurite length and branch points prior to addition of brain extracts. Buffer exchanged brain extracts were diluted 1:2 with culture medium. Half of the medium on iNs was removed (~ 100 μl) and replaced with 100 μl of 1:2 diluted buffer-exchanged extract – yielding a 1:4 diluted extract on iNs. Similarly, treatments using 1:8 and 1:16 diluted extracts were done in a similar manner. For long-term, continuous imaging, images of four fields per well were acquired every 2 h for 3 days (starting at iN day 21). Whole image sets were analyzed using Incucyte Zoom 2016A Software (Essen Bioscience, Ann Arbor, MI). The analysis job Neural Track was used to automatically define neurite processes and cell bodies based on phase contrast images. Typical settings were: Segmentation Mode - Brightness; Segmentation Adjustment - 1.2; Cell body cluster filter - minimum 500 μm^2^; Neurite Filtering - Best; Neurite sensitivity - 0.4; Neurite Width - 2 μm. Total neurite length (in millimeters) and number of branch points were quantified and normalized to the average value measured during the 6 h’ period prior to sample addition. Total neurite length is the summed length of neurites that extend from cell bodies, and number of branch points is the number of intersections of the neurites in image field.

### Size exclusion chromatography (SEC)

Whole TBS extracts (250 μl) or their void volume SEC fractions (500 μl) were injected into either a Superdex 75 (10/30HR) column or a Superdex 200 (10/300GL) column (GE Healthcare) and eluted at a flow rate of 0.8 ml/min with 50 mm ammonium acetate, pH 8.5. The 1 ml fractions were collected; 0.5 ml of this material was used for ELISA, and the other 0.5 ml was lyophilized and used for Western blotting [65]. Samples were electrophoresed on 26-well, 4–12% polyacrylamide Bis-Tris gels using MES running buffer (Invitrogen), and proteins were transferred to 0.2 μm nitrocellulose filters, the filters microwaved, and Aβ detected using a mixture of mAbs 2G3,21F12 (each at 1 μg/ml) and 0.5 μg/ml of mAb 6E10. Membranes were rinsed and incubated for 1 h with fluorescein-conjugated goat anti-rabbit or anti-mouse IgG (1:5,000; Invitrogen), and bands visualized using a LiCor Odyssey Infrared System.

### Cerebrospinal fluid sample collection and processing

Samples were collected from the L3/L4 interspace and transferred into nonabsorbing (polypropylene) tubes. CSF samples were mixed by gently inverting three or four times and then centrifuged at 400×*g* for 10 min. The crystal-clear supernatant was removed to a polypropylene tube and centrifuged at 2,000×*g* at 4 °C for 10 min, and aliquots of the supernatant were transferred to polypropylene storage tubes and stored at − 80 °C.

### Cellular Aβ (7PA2 CM) preparations

Secreted human Aβ peptides were collected and prepared from the conditioned media (CM) of a CHO cell line (7PA2) that stably expresses human APP751 containing the V717F AD mutation [52] Cells were grown in Dulbecco’s modified Eagle’s medium (DMEM) containing 10% fetal bovine serum, 1% penicillin/streptomycin, 2 mM L-glutamine, and 200 mg/ml G418 for selection. Upon reaching ~ 95% confluency, the cells were washed and cultured overnight (~ 15 h) in serum-free medium. CM was collected, spun at 1500×g to remove dead cells and debris, and stored at 4 °C. The CM was concentrated 10-fold with a YM-3 Centricon filter [73]. The concentrated CM was pooled and aliquoted to produce a large number of identical medium samples for experiments. These concentrated 7PA2 CM aliquots were stored at − 80 °C until use. To prepare the N-terminal extension of Aβs, 7PA2 CM was first incubated with DE23 anion-exchange cellulose to bind and remove the highly charged APPs while leaving the various 4–17 kDa Aβ-immunoreactive products in solution. This APP preclearing step generated CM that could then be quantitatively immunodepleted of the NTE species with the pre-β antiserum. C8, an antiserum directed to the APP C-terminus, served as a negative control since it did not immunodeplete any of the Aβ-containing species found in 7PA2 CM.

### Preparation of synthetic Aβ, including S26C dimers and dityrosine dimers

Synthetic Aβ(1–36), Aβ (1–37), Aβ(1–38), Aβ(1–39), Aβ(1–40), Aβ(1–42), Aβ (1–43), Aβ(1–45) and Aβ(1–46) all were purchased from AnaSpec (Fremont, CA). Synthetic Aβ(1–16) and Aβ (17–42) were purchased from rPeptide (Watkinsville, GA). Monomeric Aβ was prepared as described previously [72]. Aβ S26C-dimers were synthesized and purified using reverse-phase high performance liquid chromatography. Briefly, Aβ(1–40) was dissolved at 2 mg/ml in 50 mM Tris–HCl, pH 8.5, containing 7 M guanidinium HCl and 5 mM ethylenediamine tetraacetic acid, and incubated at room temperature overnight. The sample was then centrifuged at 16,000×*g* for 30 min and the upper 90% of supernatant applied to a Superdex 75 10/300 size exclusion column (GE Healthcare Biosciences, Pittsburgh, PA, USA), eluted at 0.5 ml/minute with 50 mM ammonium bicarbonate, pH 8.5, and absorbance monitored at 280 nm. Fractions of 0.5 ml were collected. The UV absorbance at 275 nm was determined for the peak fraction and the concentration of Aβ determined using ε_275_ = 1,361/M/cm [51]. Dityrosine cross-linked Aβ dimer ((Aβ(1–40))_DiY_) was prepared as reported previously and SEC-isolated as described above. The UV absorbance at 283 nm of the dimer peak fraction was measured and the concentration of DiY dimer determined using ε_283_ = 6,244/M/cm. (Aβ(1–40)S26C)_2_ was prepared as described previously and the dimer was SEC-isolated as outlined above. The UV absorbance at 275 nm of the (Aβ(1–40)S26C)_2_ peak was measured and the concentration of this dimer determined using ε_275_ = 2,722/M/cm .

Following collection and concentration determination, monomer and dimer peak fractions of the respective oligomer preparations were diluted to 48.5 μM and aliquots (20 μl) were immediately frozen on dry ice and then stored at − 80 °C. Once thawed, aliquots were used immediately either for characterization (using analytical SEC and electron microscopy) or as standards in the MSD or Erenna immunoassays.

### Preparation of synthetic pre-Aβ and N-terminally extended Aβ peptides

Synthetic preAβ and N-Terminally Extended Aβ preparation were similar to previously described [68]. Five variants of preAβ and NTE-Aβ were produced with extensions of 10, 20, or 30 residues from the APP sequence added to the N-terminus of Aβ_1–40_. The genes for N-terminally extended variants (by 10, 20, or 30 residues from APP) were produced through a stepwise extension of the synthetic Aβ_1–40_ by PCR using oligonucleotides with the desired extensions with *E. coli* preferred codons. Aβ_1–40_ and the N-terminally extended variants were expressed in *E. coli* (BL2 DE3 PLysS Star). The peptides were purified using ion exchange and size exclusion steps. The purity of the peptides was confirmed by SDS PAGE, RP-HPLC and MALDI-TOF mass spectrometry. Purified peptides were stored as lyophilized aliquots.

### Aβ1-x, Aβx-40 and Aβx-42 enzyme-linked immunosorbent assays (ELISA)

Assays utilized the Meso Scale Discovery (MSD) platform and reagents from Meso Scale (Rockville, MD). MULTI-ARRAY® 96 well small-spot black microplates were coated with 3 μg/ml of monoclonal antibody 266 in tris buffered saline (TBS) and incubated at RT for 18 h. Monoclonal antibody 266 recognizes the mid-region of Aβ, thus enabling detection of both N- and C-terminally heterogeneous Aβ species. Binding sites that were unoccupied were blocked in 150 μl of 5% Blocker A (MSD) in TBS containing 0.05% tween 20 (TBST) and agitated at 400 rpm for 1 h and 22 °C. Plates were washed 3 times with TBST and samples, and standards were applied in duplicate, and agitated at 400 rpm for 2 h and RT. After the capture phase, plates were washed 3 times with TBST and incubated with biotinylated antibody. To allow the detection of Aβ1-x or Aβx-42, we used biotinylated 3D6 (1 μg/ml) or 21F12 (1 μg/ml), respectively. Simultaneously, 1 μg/ml of the reporter reagent (SULFO-TAG Labelled Streptavidin) was added in ELISA diluent and incubated at RT with gentle agitation for 2 h. Finally, plates were washed 3 times with TBST and 2× MSD read buffer (150 μl per well) was applied to allow for electrochemiluminesence detection. A SECTOR imager was used to measure the intensity of emitted light, thus allowing quantitative measurement of analytes present in the samples.

### Statistical analysis

The LTP and LTD values reported throughout were measured at 60 min after the conditioning stimulus unless stated otherwise. Results were expressed as means ± SEM from at least 4 independent biological samples. Two-tailed Student’s t-test and one-way analysis of variance (ANOVA) were used to determine statistical significance throughout. For live-cell imaging experiments, samples and treatments were coded and tested in a blinded manner. Differences between groups were tested with One-way analysis of variance (ANOVA) with Bonferroni *post-hoc* tests or student’s *t*-tests (* *p* < 0.05, ** *p* < 0.01, and *** *p* < 0.001).

## Results

### Soluble AD brain extracts rich in aqueously-soluble Aβ inhibit hippocampal LTP

**Fig. 1:**
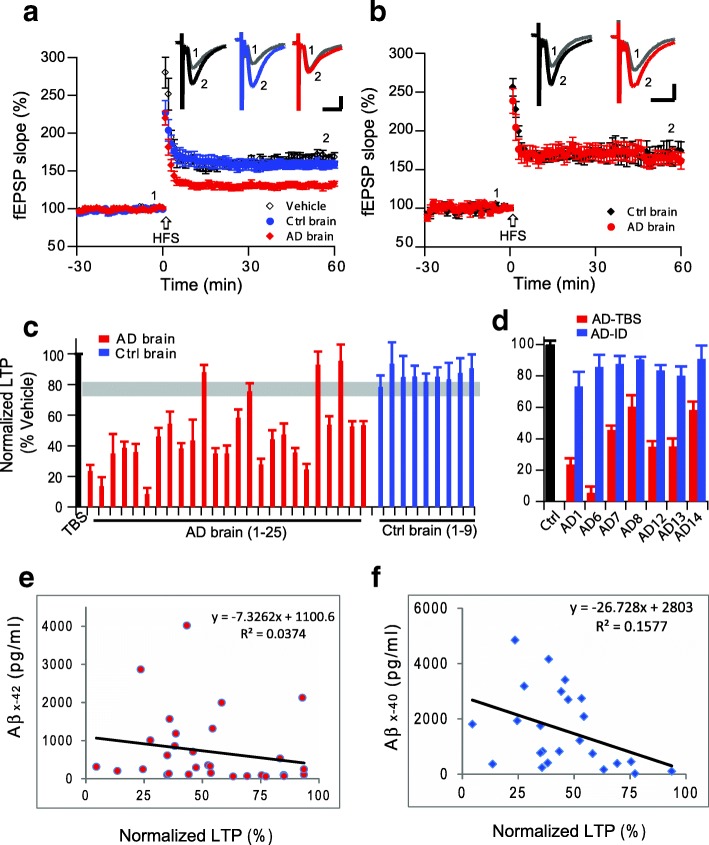
Soluble Aβ extracted by homogenization in TBS from Alzheimer’s disease brain alters hippocampal long-term potentiation. (**a**) LTP induction after treatment with vehicle (black open circles), control brain TBS extract (blue circles) and AD brain TBS extract (red diamonds) from one AD patient. (**b**) LTP after treatment with TBS extracts made from another control brain (black diamonds) or another AD brain (red circles). (**c**) Summary data of LTP results with one representative run with plain TBS buffer (black bar), 25 AD brain TBS extracts (red) and 9 control (non-AD) brain TBS extracts (blue). All LTP results represent values at 60 min post-HFS normalized to vehicle alone at that time point. Gray horizontal bar indicates the lowest LTP level from control brain. (**d**) LTP summary data of the AD TBS extracts (red) and their respective immunodepleted extracts (blue). (**e,f**) Correlations between LTP levels at 60 min and the respective [Aβ_x-42_] (**e**) and [Aβ_x-40_] (**f**) levels in the AD TBS extracts

We previously reported that soluble, human brain-derived Aβ oligomers inhibit hippocampal LTP [35, 64]. We first confirmed that some soluble Aβ-rich extracts of neuropathologically validated human (AD) cerebral cortex robustly and consistently inhibit hippocampal LTP while not altering basal synaptic transmission before the high-frequency stimulus (HFS) (control brain that were from age-matched neurologically normal human cortical extracts: 158 ± 6%, *n* = 8 vs. AD brain: 130 ± 4%, n = 8; p < 0.001) (Fig. 1a). However, cortical extracts from some other AD brains prepared using the identical procedure failed to inhibit LTP (control: 169 ± 12%, *n* = 6, vs. AD: 165 ± 9%, *n* = 7; *p* > 0.05) (Fig. 1b). Several other AD brain extracts also did not inhibit hippocampal LTP (4 out of 25, i.e. 16% of total tested AD brain. Figure 1c, red), while all non-AD brain extract did not impair LTP (Fig. 1c, blue). All clinical and neuropathological data of the AD patients and controls were listed on Additional file 1: Table S1. To verify whether the AD brain extracts that inhibited LTPs were soluble Aβ-dependent, we chose 7 AD brain extract samples that inhibited hippocampal LTP and immunodepleted their soluble Aβ by a high-titer polyclonal antiserum (AW7) to human Aβ (Additional file 1: Figure S1). Consistent with our previous finding [64], the immunodepleted AD brain extracts failed to inhibit LTP (Fig. 1d).

To further explore whether the AD brain extract-mediated impairment of LTP is dependent on the level of soluble Aβ [64], we plotted the mean LTP levels vs. the mean Aβ_x-42 and_ Aβ_x-40_ concentrations determined by specific ELISAs (Fig. 1e, f). The impairment of LTP was weakly correlated with either [Aβ_x-42_] (R^2^ = 0.04, *p* > 0.05) or [Aβ_x-40_] (R^2^ = 0.16, p > 0.05). These results suggest that although AD brain Aβ levels are significantly higher than control brain levels, LTP impairment does not correlate directly with the total brain tissue Aβ level as detected by ELISA (Fig. 1e, f). Collectively, these results confirm previous findings that Aβ per se, not other components of the AD brain extracts, are responsible for the LTP impairment, but raise the possibility that Aβ conformation or assembly state, not simply the total Aβ monomer levels, may be another factor for its bioactivity.

### Aβ oligomer-rich bioactive AD brain extracts induce neurotoxicity in iPSC-derived human neurons

**Fig. 2:**
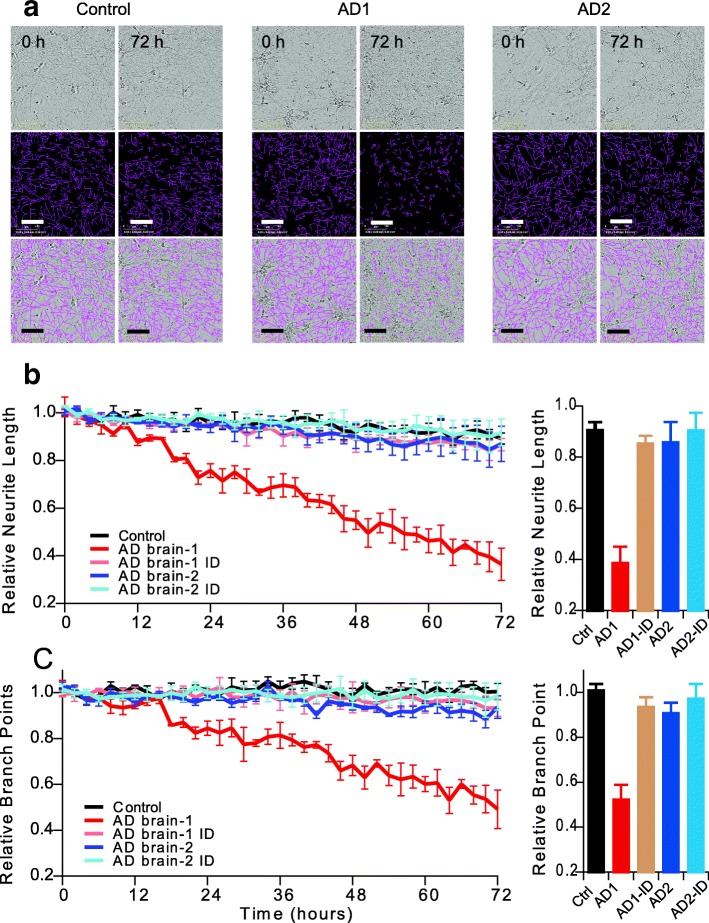
Soluble Aβ extracts that either do or do not block LTP affect human neurites accordingly. IncuCyte live-cell video microscopy monitored the effect of AD brain extracts on iPSC-derived neurogenin-induced human neurons (iNs). On post-induction day 21, iNs were treated with Control TBS (Control: black) or AD extracts (colors) and the neurons imaged for 72 h. (**a**) Phase contrast images (top panel) at 0 and 72 h were analyzed using the incuCyte NeuronTrack algorithm to identify neurites (pseudocolored pink in middle panels). Identified neurites were superimposed on the phase contrast image (bottom panel). Scale bar, 100 μm. Each well of iNs was imaged for 6 h prior to addition of the extract and NeuroTrack measured neurite length and branch points at this baseline used to normalize neurite length measured at each interval after addition of extract. (**b,c**) Time course of change in neurite length (**b**) and branch points (**c**) after addition of AD brain extracts (red and blue) or immunodepleted sample (light brown and light blue, respectively) when compare to control brain extract (black). Summary results at 72 h treatments are shown on right

To further verify whether bioactive AD brain extracts that inhibit hippocampal LTP can induce other forms of neurotoxicity, we employed a live-cell imaging paradigm using time-lapse video microscopy on an Essen IncuCyte apparatus to monitor the effects of the same AD brain extracts on iPSC-derived, neurogenin-induced human neurons [86]. Consistent with the Aβ-impaired synaptic function, a bioactive AD brain extract (Fig. 1a) also induced marked deficits of neurites as regards their length (Fig. 2b: red) and branch point number (Fig. 2c: red), both of which were reduced 50–60% compared to identical treatment with a control brain extract (Fig. 2b, c: black). Applying the Aβ-immunodepleted aliquot from this bioactive AD brain extract, the neurite length and branch point number were normal (Fig 2a; Fig. 2b, c - tan). Interestingly, when we applied a non-bioactive AD brain extract (Fig. 1b) to the iPSC neurons, neurite length and branch points remained unchanged (Fig 2a; Fig. 2b, c - blue), indicating that by both mouse hippocampal slice LTP and this human neuron assay, the inactive extract contains very low or no neurotoxic species. The Aβ-immunodepleted aliquot of this inactive AD sample was likewise inactive, as expected (Fig. 2b, c - light blue). These results suggest that Aβ-rich AD brain extracts interrupt both synaptic function and neurite intactness.

### Soluble oligomers but not other Aβ forms inhibit hippocampal LTP

**Fig. 3:**
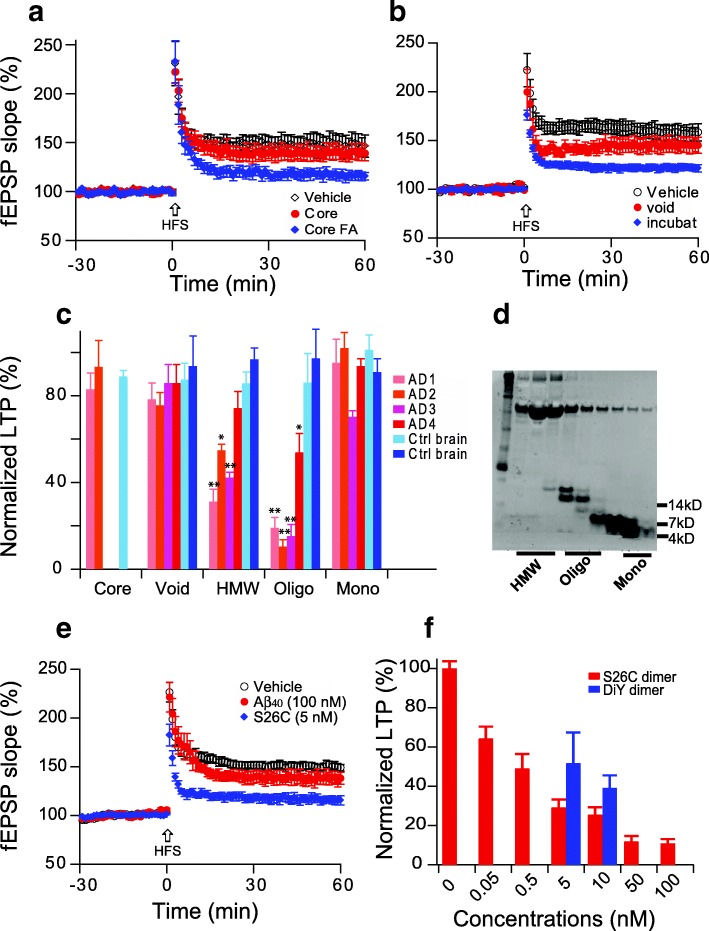
Soluble Aβ oligomers inhibit hippocampal LTP. (**a**) Insoluble amyloid plaque cores from AD cortex fail to inhibit the LTP (red), while LTP was inhibited if an equivalent aliquot of the cores was solubilized in 88% formic acid and neutralized with NaOH (blue). (**b**) The void volume fraction of a Superdex 75 SEC chromatography of an AD cortex TBS extract (red) showed no significant LTP inhibition, but incubating the void volume fraction at 37 °C for 2 days released lower MW soluble Aβ oligomers that significantly impaired LTP (blue). (**c**) Summary LTP data of the indicated AD brain fractions (*n* = 6~ 8). (**d**) Representative western blot shows different MW SEC fractions of soluble Aβ-rich AD cortical extracts; (**e**) Oxidized synthetic [Aβ_40_-S26C]_2_ dimers (blue diamonds) cause significant LTP inhibition but monomeric Aβ_1–40_ does not (red); (**f**) Dose-dependent LTP inhibition by oxidized synthetic [Aβ_40_-S26C]_2_ dimers (red), and dityrosine cross-linked Aβ1–40 dimers (DiY) (blue). *: *p* < 0.05; **: *p* < 0.01

Aβ is pleomorphic and can populate a range of assemblies, with forms ranging from the monomer all the way to the aggregates of insoluble ~ 8 nm fibrils found in amyloid plaques. In the past two decades, most studies have focused on aqueously soluble Aβ assemblies with sizes intermediate between monomer and fibrils [30]. To confirm our previous reports [64, 81], we chose four neuropathologically typical AD brains and two age-matched control brains and prepared a series of Aβ-rich extracts: insoluble amyloid plaque cores and buffer-soluble fractions separated by Superdex 75 size-exclusion chromatography (SEC) that include the void volume (> 70 kDa), high molecular weight (HMW, 17~ 60 kDa), oligomers (6~ 16 kDa) and ~ 4 kDa monomers. Consistent with earlier findings [64], the plaque core-rich extracts did not alter LTP (140 ± 8%, *n* = 6 vs. vehicle alone 147 ± 9%, *n* = 7, *p* > 0.05 (Fig. 3a), while Aβ oligomers released from the insoluble plaques by formic acid inhibited LTP (116 ± 5%, n = 7). Large soluble assemblies (void volume) from a Superdex 75 SEC column did not significantly alter LTP (145 ± 8%, *n* = 8 vs. vehicle alone: 158 ± 8%, n = 7, p > 0.05) (Fig. 3b), but when the void volume fraction was incubated at 37 °C for 2 days, the large Aβ assemblies were dissociated into smaller Aβ oligomers that could inhibit hippocampal LTP (122 ± 4%, n = 8). Figure 3c summarizes the effects on LTP of SEC fractions isolated from AD and control brain extracts, confirming that smaller soluble Aβ oligomers confer LTP impairment.

To further verify these results, we used two synthetic crosslinked Aβ dimers [51]: S26C dimers (Aβ_1–40_ with a cysteine residue in place of Ser^26^, allowing formation of disulfide crosslinked dimers) and DiY dimers (wt Aβ_1–40_, having a tyrosine at position 10, can be crosslinked to a dityrosine-linked dimer by oxidation). To assess the LTP effects of monomers and dimers, we used 100 nM Aβ_1–40_ and 5 nM [S26C]_2_ dimers. The 100 nM monomers had no effect on LTP (139 ± 7%, *n* = 7 vs. vehicle: 150 ± 4%, n = 7, p > 0.05) (Fig. 3e), while 20-fold less concentrated dimers significantly impaired LTP (116 ± 4%, *n* = 7). Figure 3f shows a dose-dependent LTP inhibition by [S26C]_2_ dimer; a similar effect can be observed with the DiY dimers.

### Soluble Aβ oligomers from other sources also inhibit hippocampal LTP

**Fig. 4:**
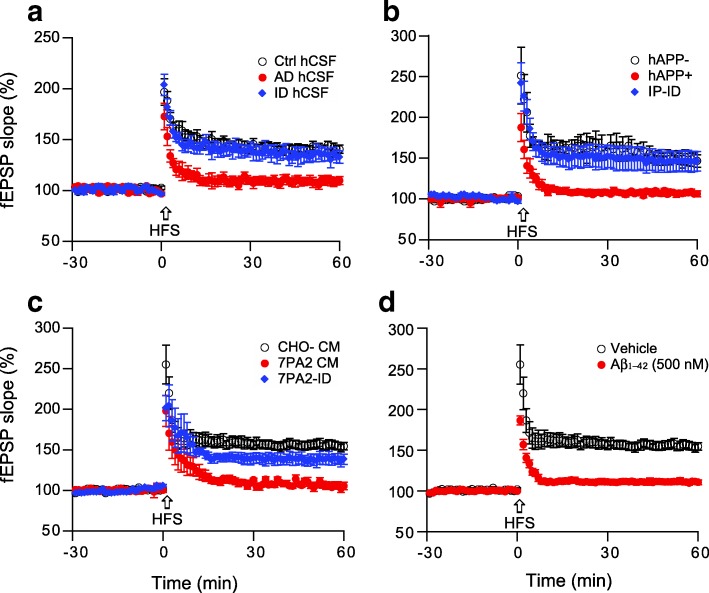
Soluble Aβ oligomers from other sources also inhibit hippocampal LTP. Several sources of soluble Aβ included (**a**) AD patient CSF; (**b**) APP tg mouse of AD (J20 mice); (**c**) cell secreted human soluble Aβ; and (**d**) synthetic Aβ_1–42_ peptide, effect on hippocampal LTP. All these impaired hippocampal LTP (red), while the inhibition of LTP by the 3 biological sources was prevented by removing soluble Aβ via immunodepletion (blue)

CSF is in direct contact with the extracellular space of the brain, and the principal species of Aβ that can be detected in CSF are aqueously soluble. To test whether the soluble Aβ from human brain plays a role in altering hippocampal LTP, we collected the CSF from the mild AD patients and concentrated them into 50 μl aliquots, then added one to the perfusion buffer (10 ml) of a mouse brain slice. LTP was significantly reduced by the AD CSF samples, while the CSF from age-matched non-AD controls produced no change vs. vehicle (Ctrl: 141 ± 4%, *n* = 6 vs. AD: 109 ± 6%, *n* = 7, *p* < 0.001) (Fig. 4a). When Aβ removed from the AD CSF by AW7 immunodepletion, hippocampal LTP was restored to the control level (135 ± 6%, *n* = 6).

To investigate whether Aβ isolated from APP tg mouse brain has an effect on LTP, we chose hAPP V717F mice generated by Mucke et al. [44], which have elevated levels of Aβ42 and Aβ42/40 ratio and develop Aβ deposits and plaque formation at 8 months of age. The brains of five 7-months-old J20 mice and 5 wild-type littermates were harvested, and Aβ extracts were prepared similarly to the AD brain extracts. The J20 but not the wild-type extracts impaired hippocampal LTP (150 ± 7%, *n* = 5 vs. 107 ± 3%, *n* = 7, *p* < 0.001), and the LTP inhibition was Aβ-dependent because immunodepleted J20 brain extract had no effect on LTP (146 ± 12%, n = 6) (Fig. 4b).

Another Aβ source is naturally secreted, soluble amyloid-β oligomers generated in a cell culture model termed 7PA2 cells, which express hAPP with the familial AD “Indiana” mutation (APPV717F). These cells secrete Aβ dimers and trimers [52] but have also been shown to secrete some N-terminally extended monomers that extend from aa − 31 to aa 40 and 42 (based on Aβ numbering) [77]. In line with other sources of Aβ, the 7PA2 CM (total Aβ 1514 ± 824 pg/ml) inhibited LTP (156 ± 5%, n = 6 vs. 106 ± 4%, n = 7, p < 0.001) (Fig. 4c). Immunodepleting Aβ prevented the inhibition of LTP to control levels (138 ± 7%, n = 7). Lastly, the widely used synthetic Aβ_1–42_ peptide (500 nM) significantly impaired hippocampal LTP (156 ± 5%, n = 6 vs. 111 ± 3%, n = 7, p < 0.001) (Fig. 4d). These results suggest that multiple sources of soluble Aβ, wherever from human or rodent and cell derived or synthetic, can specifically and significantly inhibit hippocampal LTP.

### Certain soluble Aβ fragments inhibit hippocampal LTP

**Fig. 5:**
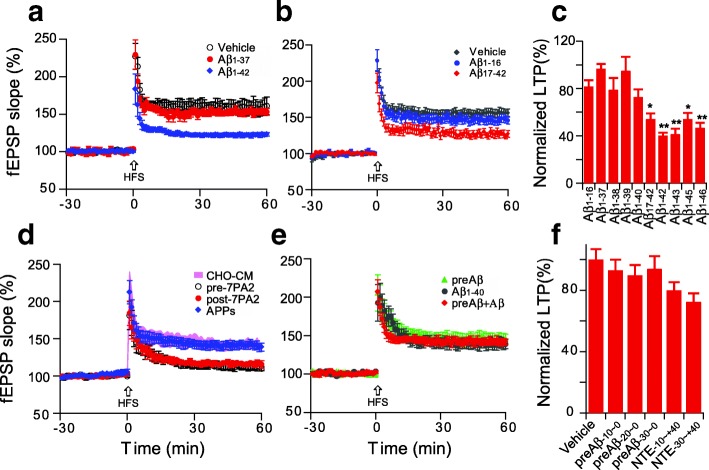
Soluble Aβ peptides with longer C-termini confer greater synaptic toxicity. (**a**) The short Aβ_1–37_ synthetic peptide did not impair hippocampal LTP at concentrations of 200 nM (red, *n* = 7, *p* > 0.05), while the same dose of the longer Aβ_1–42_ peptide showed significant inhibition (blue, n = 6, *p* < 0.001); (**b**) N-terminally truncated synthetic Aβ_1–16_ and Aβ_17–42_ effect on the hippocampal LTP. (**c**) Summary data of LTP effects of Aβ peptides of increasing lengths at 200 nM concentrations; (**d**) The whole 7PA2 CM as well as immunoprecipitated NTE-Aβs (black open circles, n = 7, p < 0.001) and the CM remaining CM after depletion of APPs by DE23 resin (red circles, n = 7, p < 0.001) all inhibit LTP, while the isolated APPs alone (blue diamonds, n = 7, p > 0.05) does not; (**e**) Treatment of slices with synthetic pre-Aβ (− 30 to − 1) does not facilitate synthetic Aβ_1–40_ to induce synaptotoxicity, that is to say, a synthetic APP-34 to − 1 fragment added to an Aβ_1–40_ peptide does not inhibit LTP (n = 6, p > 0.05); (**f**) Summary data of synthetic peptides containing or not various lengths (− 10. -20, − 30) of APP prior to the Aβ1–40 Asp1 start site (called “preAβ”) and N-terminal extension on Aβ1–40 do not inhibit LTP. (n = 6~ 8). *: *p* < 0.05; **: *p* < 0.01

Amyloidogenic processing of the amyloid precursor protein (APP) by β- and γ-secretases generates several biologically active products, including different Aβ fragments and the APP intracellular domain. It has been found that Aβ37, Aβ38, Aβ39, Aβ40, Aβ42, Aβ43 can all be detected in human cerebrospinal fluid [23, 66], while even longer, more hydrophobic Aβ peptides (Aβ45, Aβ46) can be found in cell lysate [53]. To examine the potential effects of these various species, 200 nM concentrations of Aβ_1–37_ to Aβ_1–46,_ were added to the hippocampal slice perfusate for 30 min prior to a HFS that would induce LTP. Aβ_1–37,_ Aβ_1–38_ and Aβ_1–39_ and Aβ_1–40_ peptides had little or no significant effect on LTP. Aβ_1–42,_ Aβ_1–43,_ Aβ_1–45,_ and Aβ_1–46_ each significantly inhibited LTP (Fig. 5a, c). We speculated that Aβ_1–46_ might be more potent than Aβ_1–42_ in inhibiting LTP, so we tried different concentrations of Aβ_1–46_: (200 nM, 100 nM, and 50 nM), but the degree of LTP impairment did not differ significantly (data not shown).

In addition to variable C-terminally truncated Aβ species, N-terminally truncated Aβs were also found in AD brain [6]. To assess whether the N-terminally truncated Aβs have any effect on the hippocampal LTP, we chose Aβ_1–16_ and Aβ_17–42_ to test their bioactivity on the brain slices. Consistent with short form of Aβ_1–37_, the Aβ_1–16_ (200 nM) has no significant effect on the LTP (146 ± 5%, *n* = 7 vs. 156 ± 6%, n = 7, *p* > 0.05, Fig. 5b, c). Interestingly, the Aβ_17–42_ (200 nM) has a partial effect on the LTP (127 ± 5%, n = 7 vs. 156 ± 6%, n = 7, *p* < 0.01, Fig. 5b, c), this result further suggests that the hydrophobic C-terminal of Aβ_1–42_ may initiate the Aβ aggregation to form the toxic Aβ species.

We previously reported the existence of APP proteolytic fragments released by certain cultured cells that contain the entire Aβ region plus a variable-length N-terminal extension (NTE), including a species beginning at least 34 residues N-terminal to the Asp1 start site of Aβ. We found that such NTE-Aβ monomers are secreted along with Aβ monomers and dimers by CHO cells stably expressing the FAD APP V717F mutation (called 7PA2 cells), and they contribute to the impairment of LTP by the 7PA2 CM [77]. Here, we showed that whole 7PA2 CM (0.5x, fresh made 7PA2 CM or called pre-7PA2; the total Aβs concentration by ELISA is 757 pg/ml) and the CM after depleting it of secreted APPs-α with DE23 resin (called post-7PA2) blocked LTP, while the APPs fraction itself did not alter LTP (Fig. 5d). In order to characterize certain pre-Aβ (the APP fragments before the Aβ starting point of APP_672_) fragments of APP (− 30 to − 1, − 20 to − 10, − 10 to − 10) and also Aβ species having such N-terminal extensions (− 30 to + 40, and − 10 to + 40), we applied these peptides at 1 μM concentrations; the preAβs and short form NTEs did not inhibit LTP (Fig. 5e, f). Then, we combined the preAβ_-20 to − 1_ peptide with the Aβ_1–40_ peptide; the mixed peptides did not cause Aβ_1–40_ to induce synaptic dysfunction (Fig. 5e). These results suggest that pre-Aβ, Aβ_1–16_, Aβ_1–37_, Aβ_1–39_ and NTE (− 30 to + 40), all of which are relatively more hydrophilic than the synaptotoxic Aβ_1–42,_ are not synaptotoxic, whereas the longer forms (Aβ_1–42_ to Aβ_1–46)_ that are more hydrophobic and more prone to aggregate were toxic species.

### Antibodies to the N-terminus of Aβ prevent the LTP inhibition

**Fig. 6:**
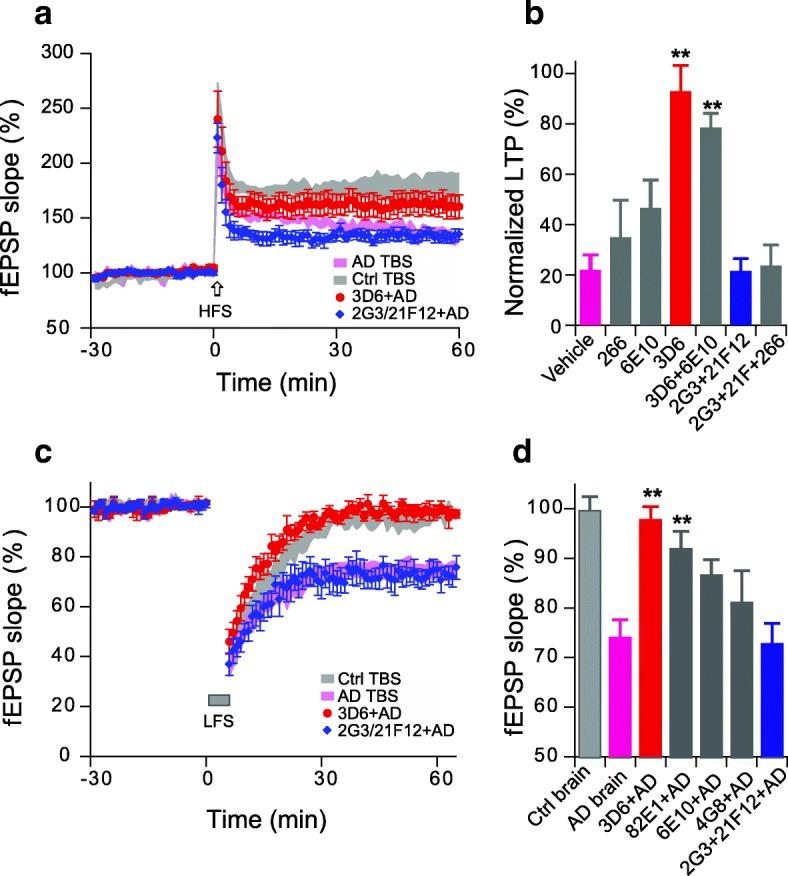
N-terminal antibodies prevent interruption of hippocampal synaptic plasticity by soluble Aβ-rich brain extract. (**a**) Asp1 N-terminus specific Aβ antibody, 3D6 (red circles), not the C-terminal-specific antibodies 2G3 + 21F12 (blue diamonds) prevent the AD brain TBS extract (purple traces) from inhibiting LTP. (**b**) Summary data of antibodies to different Aβ epitopes as to their effect on AD brain TBS extract impairment of LTP (*N* > 6 recordings for each antibody). (**c**) N-terminal antibody 3D6 (red circles), not the C-terminal target antibodies, 2G3 + 21F12 (blue diamond) prevent the AD brain extract (purple traces) from facilitating LTD. (**d**). Summary data of antibodies to different Aβ epitopes as to their effect on the AD brain extract’s facilitation of LTD (*N* > 5 recordings for each antibody). *: *p* < 0.05; **: *p* < 0.01

Active immunization with synthetic Aβ peptides or passive infusions of humanized anti-Aβ monoclonal antibodies has been widely investigated as an immunotherapeutic approach to treat and prevent clinical AD. To examine this approach in tightly-controlled, reductionist experiments, we conducted a series of studies of hippocampal LTP and LTD in the absence or presence of antibodies raised to various human APP sequences within and flanking the Aβ region. The antibodies and their epitopes were 3D6 (requires a free Asp1 at the Aβ N-terminus), 82E1 (recognizes a free Asp1 at the Aβ N-terminus), 6E10 (to aa 3–8), 266 (to aa 13–28), 4G8 (to aa 17–24), 2G3 (requires a free Gly40 at the end of aa 33~ 40), 21F12 (requires a free Ile42 at the end of aa 33~ 42) and polyclonal antibody R1282 (principally recognizes epitopes in the N-terminal third of Aβ). To test the effects of these antibodies in rescuing Aβ-impaired LTP, we first tested each antibody (at 1–3 μg/ml) itself whether has any effect on the hippocampal LTP (Additional file 1: Figure S2), then added each antibody to an AD brain extract previously shown to inhibit LTP and incubated with mixing for 30 min before adding the mixture to the slice perfusion buffer. The N-terminal antibody 3D6 but not the C-terminal antibodies 2G3 and 21F12 fully prevented LTP impairment by the soluble Aβ oligomer-rich AD extract (Fig. 6a, b). These results further supported that the N-terminally, not those C-terminally targeted antibodies of Aβ could neutralize Aβ-mediated synaptotoxicity and reduce the pathology of AD model mice [64, 83].

Another feature of synaptic plasticity is represented by long-term depression (LTD), an electrophysiological response known to correlate with dendritic spine shrinkage and synapse collapse [4, 78]. It has become widely used to assess Aβ-induced synaptic dysfunction after we first demonstrated that soluble Aβ oligomers of several sources could facilitate a persistent LTD upon a weak low-frequency (1 cps × 300 s) stimulation that usually fails to induce LTD [10, 16, 34, 38, 50, 56]. Again, two Asp1 N-terminus-specific antibodies, 3D6 and 82E1, but not the C-terminal antibodies 2G3 and 21F12, prevented the Aβ-facilitated LTD (Fig. 6c, d). A broad N-terminal region antibody, 6E10, and a mid-region antibody, 4G8, only partially prevented the Aβ-facilitated LTD. These results further support that the N-terminus of Aβ_1–42_ plays a crucial role in triggering synaptic dysfunction.

## Discussion

Here, we have performed a systematic comparative analysis of numerous features of the well-known inhibition of hippocampal synaptic plasticity by soluble oligomers of human Aβ. Our results complement and extend previous studies from our and other laboratories that found that soluble Aβ oligomers inhibit hippocampal LTP and induce synapse and neurite loss, including by oligomers isolated directly from AD cortex. We show here that Aβ inhibition of LTP is not conferred by monomers but rather by soluble oligomer-rich preparations. We also found that APP proteolytic fragments just prior to the Aβ N-terminus, the short Aβ peptides Aβ_1–16_ through Aβ_1–40_ and the N-terminally extended peptide − 30 to Aβ40 (APP641–711 of APP_770_) all failed to significantly inhibit LTP, while the longer, more hydrophobic peptides Aβ_1–42_ through Aβ_1–46_ were synaptotoxic. Two different N-terminal antibodies recognizing the free Asp1, 3D6 and 82E1, but not two C-terminal antibodies, could prevent Aβ oligomer impairment of LTP.

Cerebral Aβ accumulation and aggregation are early pathological events in AD, starting some 2 to 3 decades or more before the onset of readily detectable clinical symptoms [5, 18, 19, 67]. Although insoluble, fibrillar aggregates of Aβ constitute the neuropathological hallmark of AD, the number of plaques and the levels of insoluble Aβ correlate weakly with the local extent of synaptic and neuronal loss and thus with cognitive impairment in humans [45, 47]. On the other hand, the levels of soluble assemblies (oligomers) of Aβ appear to correlate better with disease progression in both rodent models and AD subjects [8, 32, 39, 64]. In AD model mice, spatial learning and memory have been shown to be impaired in nearly all models of Aβ overproduction by transgenic APP expression, and the onset of the cognitive decline occurred close to that of brain amyloid deposition [76]. Despite the genetic evidence suggesting a pivotal role for Aβ, considerable controversy still exists about its precise pathogenic role in AD [46]. This may be due to a lack of reliable approaches for the early AD diagnosis. For example, a sensitive method to detect the Aβ oligomers and its conformations can clarify this controversy, as some Aβ oligomer-detecting techniques (such as sodium dodecyl sulfate (SDS) polyacrylamide gel electrophoresis (PAGE)) may be relatively insensitive or even lead to artifacts [75]. The fact that brain Aβ levels measured by largely monomer-specific ELISAs did not correlate closely with degree of synaptotoxicity is not surprising; in contrast, special immunoassays that selectively detect soluble Aβ oligomers can readily distinguish extracts of AD vs. age-matched normal brain [14, 59, 74, 82].

The amyloid plaques consist of different variants of the Aβ peptide, with the most abundant being Aβ_1–40_ and Aβ_1–42_. AD brain specimen analyses have revealed numerous N-terminal or C-terminal truncated or modified Aβ species in addition to the full-length Aβ_1–40_ and Aβ_1–42_ [17, 37, 54, 55]. The sequential hydrolysis of APP by ɣ-secretase in AD generates a step-wise series of Aβ peptides terminating in residues 49, 48, 46, 45, 43, 42, 40, 39, 38 and 37 [53, 69]. Here we found that synthetic Aβ fragments longer than 1–40, including Aβ_1–42_, Aβ_1–43_, Aβ_1–45_ and Aβ_1–46_, confer significant impairment of LTP, while the shorter forms of Aβ_1–16_, Aβ_1–37_, Aβ_1–38,_ Aβ_1–39 and_ Aβ_1–40_ have little effect on synaptic function. It is generally accepted that Aβ_1–42_ plays a more pivotal role in AD pathogenesis than Aβ_1–40_ because of its much higher aggregation propensity and thus neurotoxicity [58]. It has been considered that the C- terminal hydrophobic residues in Aβ_42_ are a driving force for protein misfolding and self-assembly, leading to stabilization of neurotoxic low-order oligomers (dimers and larger) [1, 48, 83]. One report showed that Aβ_1–42_ and Aβ_1–43_ were selectively deposited principally in senile plaques while shorter Aβ peptides such as Aβ1–37, 1–38, 1–39, and Aβ1–40 were deposited more in leptomeningeal blood vessels [22]. The longer forms of Aβs likely have a greater propensity to aggregate into oligomers and bind to the cell membrane because of their longer hydrophobic C-termini residues. And the shorter forms have been shown in some studies to have anti-amyloidogenic properties [33].

In addition to the above C-terminally heterogeneous Aβ species, N-terminally extended Aβ (NTE-Aβ) monomers with up to 9 residue extensions also have been detected in human plasma [24]. We previously reported that the CM of 7PA2 cells (CHO cells expressing the hAPP V717F FAD mutation) that is rich in NTE-Aβ variants as well as Aβ monomers and dimers could block hippocampal LTP. Immunodepletion of the NTE monomers with a pre-A*β* region polyclonal antibody prevented the 7PA2 CM-mediated LTP impairment, and removing all Aβ species with a pan-Aβ antiserum R1282 prevented the LTP impairment, suggesting that the monomeric N-terminally extended APP fragments containing the entire Aβ region can impair LTP [77].

To further compare the effects of N-termini vs. C-termini of Aβ on synaptic plasticity, we used several epitope-specific antibodies. We found that antibodies to the N-terminus starting with a free Asp1 (3D6 and 82E1) could fully rescue the impaired LTP and enhanced LTD caused by AD-TBS extracts, while two C-terminal antibodies (2G3 and 21F12) had no effect. The mid-region targeted antibodies (6E10, 266 and 4G8) had minor effects that did not reach significance. These results confirm previous reports including ours that antibodies which target the N-terminus, but not those targeting the C-terminus, can neutralize Aβ-mediated synaptotoxicity and reduce Aβ plaque load [3, 64, 83], thus leading to most anti-Aβ antibodies in clinical trials targeting the N-terminus or mid-region. However, several earlier studies using C-terminal antibodies, deglycosylated antibodies (D-2H6) and naturally occurring auto-antibodies against Aβ (NAbs–Aβ), were reported to significantly reduce Aβ burden and reverse cognitive deficits in AD mouse model [11, 25, 79]. The differential effects of N-terminal vs C-terminal anti-Aβ antibodies may be due to experimental paradigm differences in AD mouse models, such as dosing, administration mode or ages of treatment. The hydrophobic C-terminus of Aβ_42_ which forms part of the core structure of the aggregates is then inaccessible to antibodies, while the N-terminus is exposed and is considered an avenue neurotoxic neutralization [48, 83, 85]. Therefore, C-terminal antibodies may be more active in the preclinical stages of AD, by binding to low aggregated (more soluble) Aβ oligomers, whereas N-terminal antibodies may better access highly aggregated Aβ oligomers and fibrils (associated with a later disease state).

Although others find the seeding activity of AD brain-derived Aβ to be at least 100 times more potent than that of Aβ from CSF or synthetic Aβ in young APP transgenic mice, we find that all sources inhibit hippocampal LTP in brain slices [12, 40]. Together with the fact that C-terminal Aβ antibodies did not rescue the Aβ-impaired synaptic function in vitro, while C-terminal antibodies could improve cognitive function in APP Tg mice [11, 25, 79]. Such discrepancies may be due to the experimental paradigm. Our experimental conditions in slice LTP studies are reductionist compared to the complexities of Aβ production, aggregation and clearance that are dynamic in vivo, as well as effects of the blood-brain barrier and Aβ-degrading enzymes which are not considered in hippocampal slices. These factors will need to be addressed in further in vivo LTP studies to verify our conclusions. Another possibility is the different readout: the synaptic plasticity in present study vs. the behavior or cerebral β-amyloidosis in others. Additional specific functional biomarker (i.e. integrative EEG, event-related potentials and oscillations) that correlated with cognition or β-amyloidosis will clarify the present conclusion.

Unfortunately, several antibodies targeting Aβ have failed in clinical trials, including bapineuzumab [57], a humanized monoclonal antibody directed against the N-terminus of Aβ that recognizes the amyloid beta 1–5 region [41], similar to murine monoclonal antibody 3D6. The reasons are likely attributable to its very low dosing in the trials due to the first appearance in AD immunotherapy of amyloid-related imaging abnormality-edema (ARIA-E). The relatively late symptomatic stage (mild-moderate AD) of subjects in this and other antibody trials could also contribute to a failure to significantly slow cognitive decline. More recent trials that began treating at early or very early symptomatic stages of AD and used substantial doses of N-terminally-directed antibodies appear to clear amyloid plaques and lead to some apparent slowing of cognitive decline [61, 63]. Our results indicated that preventing soluble Aβ oligomer formation and targeting their N-terminal residues with antibodies could be an attractive combined therapeutic approach.

## Conclusions

In this study, we have performed a systematic comparative analysis of numerous features of the well-knowninhibition of hippocampal synaptic plasticity by soluble oligomers of human Aβ. Our results provide evidence that preventing soluble Aβ oligomer formation and targeting their N-terminal residues with antibodies could be an attractive combined therapeutic approach.

## Additional file

Additional file 1: **Table S1.** Demographic and pathological data on brain samples. **Figure S1.** Characterization of AD brain extracts used for LTP experiments. (a) Half milliliter aliquots of mock immunodepleted (AD) and AW7 immunodepleted (ID-AD) extracts were analyzed by IP/WB. AW7 was used for IP and a combination of 2G3 and 21F12 was used for WB. To enable comparison 2 and 5 ng of Aβ1–42 peptide was also electrophoresed on the gel. IP/WB analysis allows the capture of Aβ structures under native conditions and their detection following denaturing SDS-PAGE. (b) The same samples were also analyzed by an MSD-based Aβx-42 immunoassay. Since GuHCl effectively disaggregates high molecular weight Aβ species, samples were analyzed with and without incubation in denaturant. Analysis of samples in the absence of GuHCl allows the measurement of native Aβ monomer, whereas, analysis of samples treated with GuHCl allows detection of disassembled aggregates. The AD extracts contained much larger amounts of aggregates than monomer, and both monomer and aggregates were effectively removed by AW7 immunodepletion. The experiments shown are typical of at least 3 separate experiments. **Figure S2.** Bath application of anti-Aβ antibodies had no significant effect on hippocampal LTP. Each data in this graph was average of at least 6 recordings. (DOCX 466 kb)
