# Supplementary material for: Decoding the synaptic dysfunction of bioactive human AD brain soluble Aβ to inspire novel therapeutic avenues for Alzheimer’s disease
Source: Acta Neuropathol Commun. 2018 Nov 8;6:121. doi: 10.1186/s40478-018-0626-x (PMC6225562; doi:10.1186/s40478-018-0626-x)
Supplement: Supplementary file 1 — Table S1. Demographic and pathological data on brain samples. Figure S1. Characterization of AD brain extracts used for LTP experiments. (a) Half milliliter aliquots of mock immunodepleted (AD) and AW7 immunodepleted (ID-AD) extracts were analyzed by IP/WB. AW7 was used for IP and a combination of 2G3 and 21F12 was used for WB. To enable comparison 2 and 5 ng of Aβ1–42 peptide was also electrophoresed on the gel. IP/WB analysis allows the capture of Aβ structures under native conditions and their detection following denaturing SDS-PAGE. (b) The same samples were also analyzed by an MSD-based Aβx-42 immunoassay. Since GuHCl effectively disaggregates high molecular weight Aβ species, samples were analyzed with and without incubation in denaturant. Analysis of samples in the absence of GuHCl allows the measurement of native Aβ monomer, whereas, analysis of samples treated with GuHCl allows detection of disassembled aggregates. The AD extracts contained much larger amounts of aggregates than monomer, and both monomer and aggregates were effectively removed by AW7 immunodepletion. The experiments shown are typical of at least 3 separate experiments. Figure S2. Bath application of anti-Aβ antibodies had no significant effect on hippocampal LTP. Each data in this graph was average of at least 6 recordings. (DOCX 466 kb) [file 40478_2018_626_MOESM1_ESM.docx]

# **Decoding the synaptic dysfunction of bioactive human AD brain soluble Aβ to inspire novel therapeutic avenues for Alzheimer’s disease**

Shaomin Li^1^, Ming Jin^1^, Lei Liu^1^, Yifan Dang^1^, Beth L. Ostaszewski^1^, Dennis J. Selkoe^1^

Department of Neurology, Ann Romney Center for Neurologic Diseases, Brigham and Women’s Hospital and Harvard Medical School, 60 Fenwood Road, Boston, MA 02115, USA

**Additional file 1: Table S1- Demographic and pathological data on brain samples**

| Patients | Age (yrs) | Gender | Clinical presentation | Neuropathological diagnosis |
| --- | --- | --- | --- | --- |
| AD1 | 81 | Female | AD | AD |
| AD2 | 92 | Female | AD | AD |
| AD3 | 78 | Male | Normal pressure hydrocephalus | AD |
| AD4 | 82 | Male | AD | AD |
| AD5 | 73 | Female | Dementia | AD |
| AD6 | 68 | Male | Dementia | AD |
| AD7 | 60 | Male | Down syndrome, AD | AD |
| AD8 | 85 | Female | Dementia | AD |
| AD9 | 72 | Male | AD | AD |
| AD10 | 70 | Female | AD | AD |
| AD11 | 82 | Female | AD | AD |
| AD12 | 91 | Female | AD | AD |
| AD13 | 81 | Male | AD | AD |
| AD14 | 78 | Male | AD | AD |
| AD15 | 77 | Female | AD | AD |
| AD16 | 86 | Male | AD | AD |
| AD17 | 83 | Male | AD | AD |
| AD18 | 92 | Female | AD | AD |
| AD19 | 69 | Male | AD | AD |
| AD20 | 75 | Male | AD | AD |
| AD21 | 87 | Female | AD | AD |
| AD22 | 79 | Male | AD | AD |
| AD23 | 82 | Male | AD | AD |
| AD24 | 86 | Female | AD | AD |
| AD25 | 76 | Female | AD | AD |
|  |  |  |  |  |
| Ctrl 1 | 92 | Male | CJD | Lewy Body Dementia |
| Ctrl 2 | 34 | Female |  | Leukodystrophy |
| Ctrl 3 | 79 | Female | Recent onset of confusion and agitation | Paraneoplastic cerebellar degeneration |
| Ctrl 4 | 75 | Female |  | colloid cyst of 3rd ventricle |
| Ctrl 5 | 75 | Male | Recent onset of confusion | Stroke |
| Ctrl 6 | 69 | Male | Dementia | Multi-infarct dementia |
| Ctrl 7 | 52 | Male |  | Ventriculomegaly |
| Ctrl 8 | 70 | Female | Atypical dementia | Frontotemporal atrophy |
| Ctrl 9 | 64 | Male | CJD | Cerebellar degeneration |

**Additional file 1: Figure S1**

**Figure S1. Characterization of AD brain extracts used for LTP experiments.** **(a)** Half milliliter aliquots of mock immunodepleted (AD) and AW7 immunodepleted (ID-AD) extracts were analyzed by IP/WB. AW7 was used for IP and a combination of 2G3 and 21F12 was used for WB. To enable comparison 2 and 5 ng of Aβ1-42 peptide was also electrophoresed on the gel. IP/WB analysis allows the capture of Aβ structures under native conditions and their detection following denaturing SDS-PAGE. **(b)** The same samples were also analyzed by an MSD-based Aβx-42 immunoassay. Since GuHCl effectively disaggregates high molecular weight Aβ species, samples were analyzed with and without incubation in denaturant. Analysis of samples in the absence of GuHCl allows the measurement of native Aβ monomer, whereas, analysis of samples treated with GuHCl allows detection of disassembled aggregates. The AD extracts contained much larger amounts of aggregates than monomer, and both monomer and aggregates were effectively removed by AW7 immunodepletion. The experiments shown are typical of at least 3 separate experiments.

**Additional file 1: Figure S2**

**Figure S2. Bath application of anti-Aβ antibodies had no significant effect on hippocampal LTP.** Each data in this graph was average of at least 6 recordings.
